# Intestinal epithelial CGAS dampens inflammation by upregulating autophagy

**DOI:** 10.1080/27694127.2022.2098305

**Published:** 2022-07-19

**Authors:** Elizabeth A. Novak, Heather L. Mentrup, Nazih Bizri, Anna E. Ramos, Kevin P. Mollen, Sidrah Khan

**Keywords:** Beclin 1, colitis, IBD, LC3, macroautophagy

## Abstract

The pathogenesis of intestinal inflammation involves a complex network of cell signaling pathways. CGAS (cyclic GMP-AMP synthase) is a cytoplasmic DNA sensor that is most known for upregulating interferon-mediated inflammation. In this punctum, we discuss our novel finding that in the intestinal epithelium, CGAS binds to BECN1 (beclin 1) to subsequently induce macroautophagy/autophagy and dampen inflammation.

The seemingly endless complexity of the pathogenesis of inflammatory bowel disease (IBD) has revealed that disease variability is likely due, in part, to the intrinsic heterogeneity of the affected individuals. A multitude of pro-inflammatory signaling pathways have been demonstrated to contribute to the inflammatory processes of IBD, highlighting that a “one-size-fits-all” approach will not work for the treatment of patients. It has been known for some time that signaling pathways in the innate immune system play crucial roles in driving intestinal epithelial inflammation during IBD. More recently, the importance of the intestinal epithelial cells (IECs) themselves to the mucosal immune response of IBD has begun to be appreciated. In our paper we studied the role of cell signaling and autophagy in intestinal epithelial cells during inflammation in order to better understand the pathogenesis of IBD [1].

Autophagy is a crucial protective mechanism during which damaged organelles are sequestered within double-membrane vesicles called autophagosomes. These autophagosomes then fuse with lysosomes to degrade the damaged proteins. In our study, we outlined a novel pathway by which the DNA sensor CGAS upregulates autophagy to dampen inflammation and prevent intestinal epithelial cell death. CGAS is a cytoplasmic DNA-sensing receptor most commonly known for converting ATP and GTP into 2’-3’ cyclic GMP-AMP (cGAMP). cGAMP then serves as a secondary messenger to upregulate STING1 (stimulator of interferon response cGAMP interactor 1) and subsequent type 1 interferons. Many studies have demonstrated important roles for CGAS in inflammatory disease processes; however, a role for CGAS in IBD has yet to be elucidated. Our goal for this study was to understand the role of CGAS in the modulation of intestinal inflammation during IBD.

To understand the importance of CGAS within the intestinal epithelium of patients with IBD, we first assessed the *CGAS* gene expression and protein levels in the colonic epithelium of patients with active ulcerative colitis (UC). Our data demonstrate an upregulation of *CGAS* gene and protein levels within the colonic epithelium of patients with UC compared to control patients. We next subjected WT mice to an acute intestinal injury model. This model involves treating mice with dextran sodium sulfate (DSS, 2%) in their drinking water for 7 days. At the conclusion of the model, we found increased levels of *Cgas* gene and protein levels in their IECs, similar to what we found in human UC. To further understand the role of CGAS in intestinal inflammation, we subjected *cgas* KO mice to a similar model of experimental colitis. Interestingly, we found that even though CGAS is upregulated in the intestinal epithelium of humans with UC and WT mice subjected to experimental colitis, *cgas* knockout mice exhibit worse intestinal inflammation as demonstrated by increased weight loss, higher disease activity index scores, shorter colon lengths, increased intestinal architecture distortion, and higher levels of pro-inflammatory cytokines as compared to WT mice. This led us to hypothesize that CGAS is likely upregulated during intestinal inflammation as a part of a homeostatic mechanism in response to an inflammatory insult.

To further delineate the mechanism by which CGAS mitigates intestinal inflammation, we investigated whether CGAS may play a role in epithelial autophagy. Previous literature has demonstrated that CGAS directly binds to BECN1, a protein initiates and upregulates autophagy in fibroblasts and bone marrow-derived macrophages. Our data demonstrated that during DSS colitis, BECN1 is upregulated in the IECs of WT mice. No such upregulation is seen in the *cgas* KO mice. Furthermore, we showed an upregulation of MAP1LC3/LC3, a protein that contributes to the maturation of the autophagosome, in the colonic epithelium of WT mice. In order to further elucidate the underlying mechanism, we utilized HCT-116 cells, a human colon adenocarcinoma cell line. We transfected a plasmid encoding GFP-CGAS into HCT-116 cells and demonstrated direct binding between CGAS and BECN1 via co-immunoprecipitation. To measure autophagic flux after transfection of *CGAS*, we stimulated HCT-116 cells with poly(dA: dT) and treated them with chloroquine. Our data showed that transfection of *CGAS* leads to an increase in autophagic flux in HCT-116 cells as demonstrated by an increased LC3-II:I ratio. To determine if these findings can be recapitulated in mouse tissue, we isolated IECs from *cgas* KO and WT mice after they were subjected to DSS colitis. We treated these cells with chloroquine and measured protein levels of LC3-I and -II. Our data showed that in the IECs isolated from WT mice subjected to DSS colitis, there is an increase in autophagic flux, whereas this is not seen in *cgas* KO mice subjected to DSS colitis.

To understand how this information could be of clinical benefit to patients with IBD, we wanted to determine whether a rescue of autophagy could ameliorate intestinal inflammation. Rapamycin is a known autophagy inducer and benefits a small cohort of patients with refractory IBD. We treated *cgas* KO and WT mice subjected to DSS colitis with rapamycin. Rapamycin treatment increases protein levels of LC3-II in the colonic tissue of both WT and *cgas* KO mice. Similarly, rapamycin increases autophagic flux and the LC3-II:-I ratio in HCT-116 cells regardless of whether an empty vector plasmid or *CGAS* plasmid is transfected. Interestingly, rapamycin significantly decreases intestinal inflammation in CGAS-deficient mice to match that of the WT mice subjected to DSS. However, rapamycin treatment does not affect the disease severity of WT mice. From these data, we concluded that rapamycin can induce autophagy within the intestinal epithelium and that this induction can dampen inflammation. However, this rapamycin-driven increase in autophagy likely only occurs in the setting of predisposed autophagic dysfunction. This may explain why rapamycin has only shown efficacy in a small cohort of patients with medically refractory IBD.

Our study demonstrated a novel pathway by which CGAS upregulates autophagy within the intestinal inflammation, whereas a disruption of this homeostatic pathway leads to worsened intestinal disease. Understanding the importance of precision medicine, with further investigation we hope to show the potential benefits of utilizing CGAS as a biomarker for dysfunctional autophagy. Identifying which patents have either diminished or dysfunctional autophagy can lead to these patients receiving targeted therapy to improve their colitis severity and quality of life.
